# Trajectories of depressive symptoms and associated patterns of cognitive decline

**DOI:** 10.1038/s41598-020-77866-6

**Published:** 2020-11-30

**Authors:** Tomáš Formánek, Zsófia Csajbók, Katrin Wolfová, Matěj Kučera, Sarah Tom, Dag Aarsland, Pavla Cermakova

**Affiliations:** 1 National Institute of Mental Health, Klecany, Czech Republic; 2 Third Faculty of Medicine, Charles University Prague, Ruská 87, 100 00 Prague 10, Czech Republic; 3 Departments of Neurology and Epidemiology, Columbia University, New York, USA; 4 Department of Old Age Psychiatry Institute of Psychiatry, Psychology and Neuroscience, King’s College London, London, UK; 5 Centre of Age-Related Medicine, University Hospital Stavanger, Stavanger, Norway; 6 Second Faculty of Medicine, Charles University, Prague, Czech Republic

**Keywords:** Epidemiology, Cognitive ageing

## Abstract

The aim was to investigate the pattern and rate of cognitive decline across distinctive trajectories of depressive symptoms in older adults. In this prospective multinational cohort study on 69,066 participants (on average 64 years at baseline, 55% women), assessments of cognitive functions (immediate recall, delayed recall, verbal fluency) and depressive symptoms (EURO-D scale) were conducted at 2-year intervals. The trajectories of depressive symptoms were obtained using latent growth mixture modelling, cognitive decline was assessed using smoothing splines and linear mixed effects models. Four distinct trajectories of depressive symptoms were identified: constantly low (n = 49,660), constantly high (n = 2999), increasing (n = 6828) and decreasing (n = 9579). Individuals with increasing and constantly high depressive symptoms showed linear cognitive decline, while those with constantly low and decreasing depressive symptoms had fluctuating cognition. Participants with increasing depressive symptoms had the fastest decline, while those with decreasing symptoms were spared from decline in cognition. This study suggests that the pattern as well as the rate of cognitive decline co-occurs with specific patterns of changes in depressive symptoms over time. The most pronounced cognitive decline is present in individuals, in whom depressive symptoms increase late in life. Unique mechanisms of cognitive decline may exist for subgroups of the population, and are associated with the trajectory of depressive symptoms.

## Introduction

The number of individuals aged 60 years or more has doubled since 1980 and is expected to double again by 2050^1^. The continuously increasing life expectancy is a major success of public health, however, demographic ageing of the society brings several challenges, such as declining cognitive abilities. Even slight decrease in cognitive functions constitutes an independent risk factor for mortality^2^, impaired physical functioning and development of dementia^3^, increased health-related costs^4^ and reduced quality of life^5^. Several somatic and psychiatric conditions, as well as life-style characteristics have been linked with cognitive decline^6,7^. Among them, depression seems to be particularly closely linked with subsequent decline in cognition. Many older adults have, both, depressive symptoms and cognitive impairment^8^ and this combination seems to be in particularly associated with a higher risk of dementia^9^.

Although the exact nature of the association of depression and cognitive decline is not entirely clear, several explanations about the temporality of this association have been proposed. Studies suggest that depression may be causally linked to subsequent cognitive impairment^10^, but it may be also its consequence, specifically its prodromal phase^11^ or a psychological reaction to perceived changes in cognitive functioning^12^. Several biological mechanisms have been suggested to underlie the association between depression and cognitive impairment. Depression was suggested to exert neurotoxic effects, which is supported by evidence of reduced levels of brain-derived neurotrophic factor in suicide subjects^13^. The co-occurrence of lifetime history of depression and rapid cognitive decline is marked by increased plaque and tangle pathology in the hippocampus^10,14^, and brain microRNAs associated with late-life depressive symptoms are also associated with cognitive decline^15^. In addition, depression in older adults is frequently associated with cerebrovascular comorbidities and microvascular lesions, a concept called vascular depression^16^. The association of depression with cognitive impairment may thus be also a consequence of shared cardiovascular risk factors^16^.

Studying the relationship between trajectories of depressive symptoms and cognitive decline may enhance our understanding of the time course and directionality of the depression-cognition association. Specifically, this would enable a more dynamic view on how the deteriorating cognition over time is mirrored in longitudinal changes of depressive symptoms. A small number of studies assessed the rate of cognitive decline across different trajectories of depressive symptoms^17–19^. Using data from the English Longitudinal Study of Ageing, Zheng and colleagues identified three groups of participants based on the number of occasions when elevated depressive symptoms were present, showing that persistent depressive symptoms are associated with the highest rate of cognitive decline^18^. Based on the Korean Longitudinal Study of Ageing, Choi and colleagues employed latent class trajectory models to identify trajectories of depressive symptoms^17^. They identified four groups of participants, demonstrating that individuals with constantly high depressive symptoms have the greatest rate of cognitive decline.

Nevertheless, to our best knowledge, no studies used either considerably large or multinational samples to assess the rate of cognitive decline in distinctive depressive symptoms groups. In addition, no studies realized before included participants also from Central and Eastern Europe (CEE), which is a region previously underrepresented in research on mental health^20–22^. Moreover, studies using data-driven approaches and not a priori classifications, which are prone to high level of arbitrariness with regard to creation of groups, are scarce. In the present study, which capitalizes on data from almost 70 000 older European individuals from 18 countries, representing all regions of Europe, we aimed to use a data-driven approach based on latent class trajectory modelling to identify trajectories of depressive symptoms and then assess the rate of cognitive decline in them.

## Results

Individuals with constantly low depressive symptoms had the highest baseline cognitive functions amongst all groups, while those with constantly high depressive symptoms had the lowest cognition. Participants with increasing and decreasing depressive symptoms had a similar baseline level of all cognitive measures. Participants with constantly high depressive symptoms were the oldest (median 66 years), had the highest proportion of women (79%) and were socioeconomically the worst-off (Table 1). They had the highest number of chronic diseases and limitations. On the contrary, individuals with constantly low depressive symptoms were the youngest (median 62 years), included more men, had the highest socioeconomic status and the best health profile. Multivariable analysis (Supplementary table 1) demonstrates that when compared to individuals with constantly low depressive symptoms, those with constantly high, increasing and decreasing depressive symptoms were more likely women, socioeconomically as well as health-wise worse off.

**Table 1:** Baseline characteristics of the participants across trajectories of depressive symptoms.

	Depressive symptoms				*p* value
	Constantly low (n = 49 660; 71.9%)	Constantly high (n = 2 999; 4.3%)	Increasing (n = 6 828; 9.9%)	Decreasing (n = 9 579; 13.9%)	
Cognitive functions					
Immediate recall, mean ± SD	5.4 ± 1.7	4.2 ± 1.8	4.9 ± 1.8	4.8 ± 1.8	< 0.001
Delayed recall, mean ± SD	4.0 ± 2.0	2.7 ± 2.0	3.4 ± 2.1	3.3 ± 2.1	< 0.001
Verbal fluency, mean ± SD	21.0 ± 7.4	15.8 ± 7.2	18.6 ± 7.4	18.5 ± 7.5	< 0.001
Sociodemographic characteristics					
Age, median (IQR)	62 (14)	66 (18)	65 (16)	63 (16)	< 0.001
Woman, n (%)	24,637 (49.6)	2336 (77.9)	4537 (66.4)	6728 (70.2)	< 0.001
More than high school education, n (%)	11,919 (24.0)	253 (8.4)	1057 (15.5)	1441 (15.0)	< 0.001
Highest decile of household net worth, n (%)	5670 (11.4)	117 (3.9)	527 (7.7)	592 (6.2)	< 0.001
Living with a partner, n (%)	37,263 (75.0)	1797 (59.9)	4765 (69.8)	6123 (63.9)	< 0.001
2 and more children, n (%)	36,809 (74.1)	2097 (69.9)	4984 (73.0)	6805 (71.0)	< 0.001
2 and more grandchildren, n (%)	24,779 (49.9)	1811 (60.4)	3925 (57.5)	5351 (55.9)	< 0.001
Currently working, n (%)	22,426 (45.2)	885 (29.5)	2432 (35.6)	3440 (35.9)	< 0.001
Health-related factors					
Treatment of depression, n (%)	1409 (2.8)	788 (26.3)	590 (8.6)	1215 (12.7)	< 0.001
Body mass index, mean ± SD	26.7 ± 4.4	27.7 ± 5.6	27.3 ± 4.8	27.2 ± 5.1	< 0.001
2 and more chronic diseases, n (%)	19,519 (39.3)	2319 (77.3)	3860 (56.5)	6019 (62.8)	< 0.001
2 and more limitations in IADL, n (%)	1087 (2.2)	815 (27.2)	489 (7.2)	1146 (12.0)	< 0.001
Maximal grip strength, mean ± SD	36.4 ± 11.9	25.2 ± 10.6	30.9 ± 11.2	29.7 ± 11.3	< 0.001
Physical inactivity, n (%)	2770 (5.6)	907 (30.2)	856 (12.5)	1524 (15.9)	< 0.001
Smoking, n (%)	23,771 (47.9)	1166 (38.9)	2885 (42.3)	4248 (44.3)	< 0.001
Alcohol, n (%)	8837 (17.8)	305 (10.2)	1023 (15.0)	1330 (13.9)	< 0.001

IQR, interquartile range; SD, standard deviation; IADL, instrumental activitiy of daily living.

**Figure 1:**
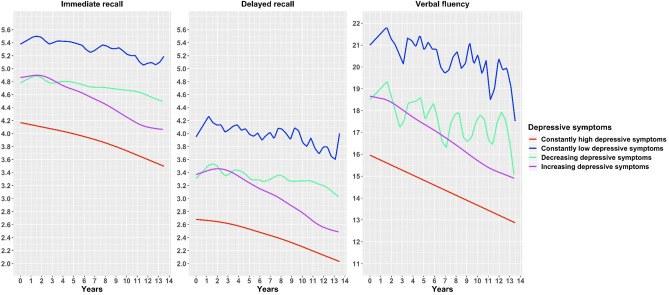
Cognitive decline across trajectories of depressive symptoms from smoothing splines models.

Using smoothing splines mixed effects models (Fig. 1), we observed four distinct trajectories of cognitive decline based on depressive symptoms. Those with constantly high and increasing depressive symptoms had a decline in cognition that showed a sign of a linear trend. On the contrary, a highly fluctuating pattern of cognitive decline was present amongst those with constantly low and decreasing depressive symptoms. Despite a similar initial level of cognitive function at baseline, the groups with increasing and decreasing depressive symptoms diverged in the rate of cognitive decline, with the group with increasing depressive symptoms demonstrating a markedly steeper cognitive decline.

**Figure 2:**
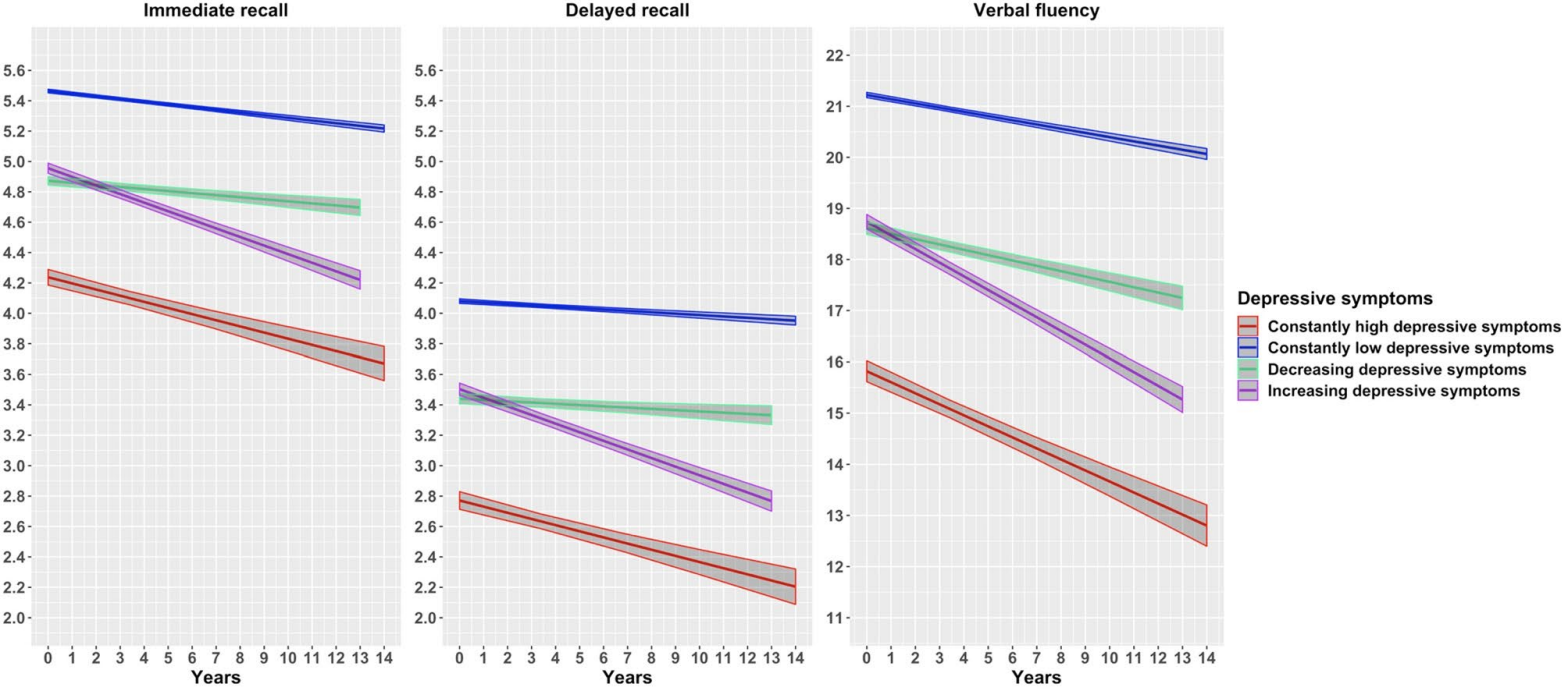
Cognitive decline across trajectories of depressive symptoms from linear mixed effects models. Results are adjusted for age, sex, education and country of origin.

**Figure 3:**
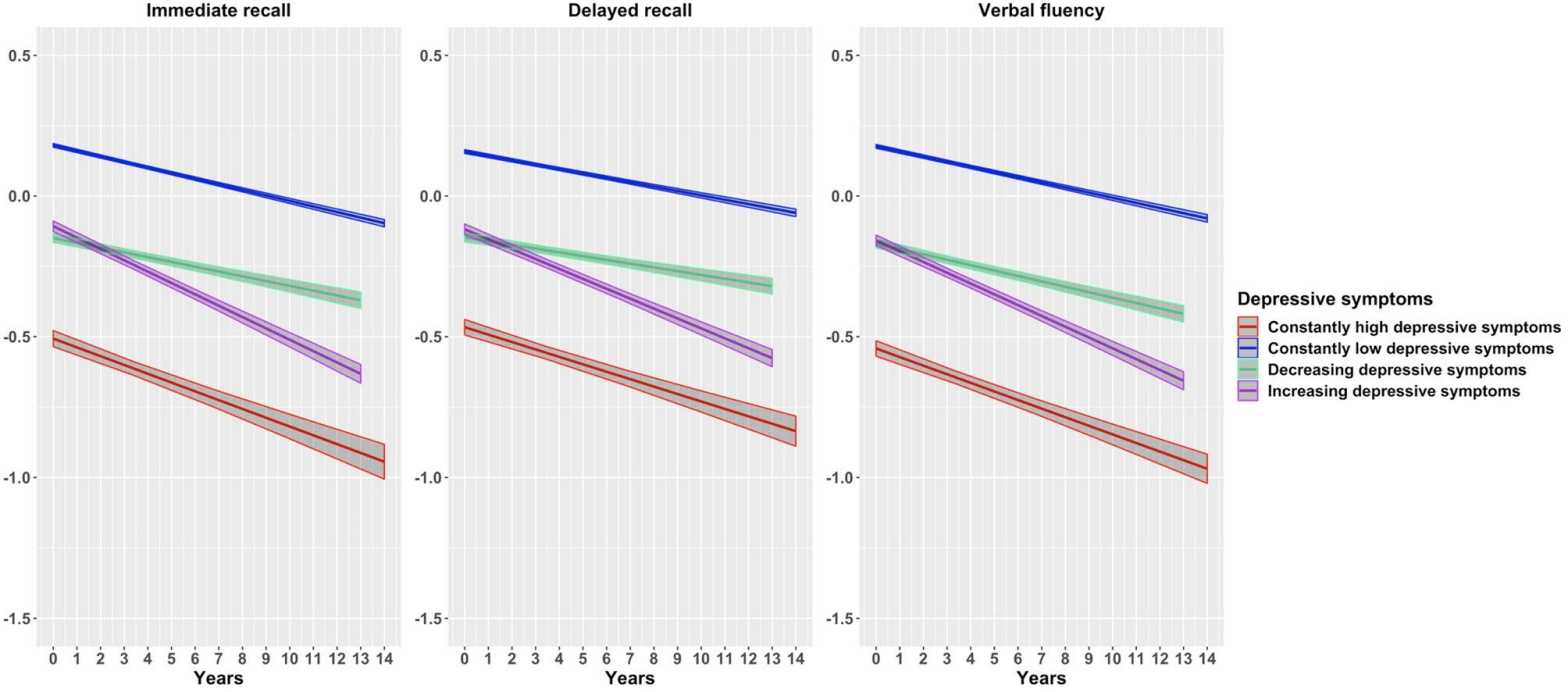
Cognitive decline across trajectories of depressive symptoms from linear mixed effects models—z-scores standardized responses. Results are adjusted for age, sex, education and country of origin.

When modelling the rate of cognitive decline using linear mixed effects models, after adjusting for age, sex, education and country of origin (Table 2, Model 1; Fig. 2), we observed that the steepest decline in all three cognitive domains was detected in individuals who had increasing depressive symptoms, followed by those with constantly high depressive symptoms. Individuals with decreasing depressive symptoms had overall the smallest magnitude of change over time and on both recall tests even no significant decline at all. Participants with constantly low depressive symptoms had a low, and in the case of delayed recall insignificant rate of decline, similar to those with decreasing symptoms. When controlling for all socio-demographic, behavioral and clinical characteristics, the rates of cognitive decline were attenuated in all groups and across all domains of cognitive functioning (Table 2, Models 2–3), but remained statistically significant in almost all cases. The only exceptions are the rates of cognitive decline on both recall tests in constantly high and on verbal fluency test in decreasing depressive symptoms groups, which were no longer statistically significant. The magnitude of cognitive decline was approximately the same across all of the three examined domains of cognitive functioning (Table 3, Fig. 3).

**Table 2:** Cognitive decline using linear mixed effects models across trajectories of depresssive symptoms.

	Depressive symptoms			
	Constantly high	Constantly low	Increasing	Decreasing
Immediate recall				
Model 1	− 0.02 (− 0.03; − 0.01)***	− 0.01 (− 0.01; − 0.01)***	− 0.04 (− 0.05; − 0.04)***	− 0.00 (− 0.01; 0.00)
Model 2	− 0.02 (− 0.03; − 0.00)*	− 0.01 (− 0.01; − 0.01)***	− 0.04 (− 0.05; − 0.03)***	− 0.00 (− 0.01; 0.01)
Model 3	0.00 (− 0.01; 0.02)	− 0.00 (− 0.01; − 0.00)*	− 0.02 (− 0.02; − 0.01)***	0.01 (0.00; 0.02)***
Delayed recall				
Model 1	− 0.03 (− 0.04; − 0.01)***	− 0.00 (− 0.00; 0.00)	− 0.05 (− 0.05; − 0.04)***	0.01 (− 0.00; 0.01)
Model 2	− 0.03 (− 0.04; − 0.01)***	− 0.00 (− 0.00; 0.00)	− 0.04 (− 0.05; − 0.04)***	0.00 (− 0.00; 0.01)
Model 3	− 0.01 (− 0.02; 0.00)	0.01 (0.00; 0.01)***	− 0.02 (− 0.03; − 0.01)***	0.02 (0.01; 0.02)***
Verbal fluency				
Model 1	− 0.16 (− 0.20; − 0.12)***	− 0.07 (− 0.08; − 0.07)***	− 0.22 (− 0.24; − 0.20)***	− 0.07 (− 0.10; − 0.05)***
Model 2	− 0.15 (− 0.19; − 0.11)***	− 0.07 (− 0.08; − 0.06)***	− 0.21 (− 0.24; − 0.19)***	− 0.07 (− 0.09; − 0.04)***
Model 3	− 0.07 (− 0.11; − 0.03)***	− 0.02 (− 0.03; − 0.01)***	− 0.12 (− 0.14; − 0.09)***	− 0.02 (− 0.04; 0.01)

Results are β (95% CI) derived from linear mixed-effects models.

*p < 0.05; **p < 0.01; ***p < 0.001.

Model 1: time (in years since baseline), age, sex, education and country of origin.

Model 2: time (in years since baseline), age, sex, education, country of origin, household net worth, current job situation, family status, number of children and grandchildren.

Model 3: time (in years since baseline), age, sex, education, country of origin, household net worth, current job situation, family status, number of children, number of grandchildren, treatment of depression, number of limitations in IADL, number of chronic diseases, body mass index, physical inactivity, smoking, alcohol use and maximal grip strength.

**Table 3:** Cognitive decline on z-score transformed cognitive domains using linear mixed effects models across trajectories of depresssive symptoms.

	Depressive symptoms			
	Constantly high	Constantly low	Increasing	Decreasing
Immediate recall	− 0.03 (− 0.04; − 0.03)***	− 0.02 (− 0.02; − 0.02)***	− 0.04 (− 0.04; − 0.04)***	− 0.02 (− 0.02; − 0.01)***
Delayed recall	− 0.03 (− 0.03; − 0.02)***	− 0.02 (− 0.02; − 0.01)***	− 0.04 (− 0.04; − 0.03)***	− 0.01 (− 0.02; − 0.01)***
Verbal fluency	− 0.03 (− 0.03; − 0.03)***	− 0.02 (− 0.02; − 0.02)***	− 0.04 (− 0.04; − 0.04)***	− 0.02 (− 0.02; − 0.02)***

The dependent variables were transformed to z-scores.

Results are β (95% CI) derived from linear mixed-effects models.

*p < 0.05; **p < 0.01; ***p < 0.001.

All models were adjusted for baseline age, sex, education and country of origin.

### Secondary analyses

When stratified by baseline age, the results in, both, younger (less than 65 years old) and older participants indicate that the group with increasing depressive symptoms is associated with the highest rate of cognitive decline in all domains of cognitive functioning (Supplementary Table 6). Similarly, when stratifying by region of residence, with the exception of Israel, in all regions, increasing depressive symptoms were associated with the most pronounced rate of cognitive decline (Supplementary Table 7). In Israel, we observed the highest rate of cognitive decline in the group with constantly high depressive symptoms. The results of Granger causality indicated that changes in cognitive functioning, consistently across all three cognitive domains, precede the changes in depressive symptoms.

## Discussion

In this community-based prospective cohort study of 70,000 well-characterized middle-aged and older individuals, we identified four distinct groups of individuals based on the patterns of depressive symptoms: (1) constantly high depressive symptoms, (2) constantly low depressive symptoms, (3) increasing depressive symptoms and (4) decreasing depressive symptoms. We found that individuals showcasing increasing depressive symptoms had the highest rate of cognitive decline, followed by the individuals with constantly high depressive symptoms. Both groups were associated with cognitive decline that followed an almost linear pattern. On the contrary, the cognitive decline in individuals with decreasing and constantly low depressive symptoms resembled a non-linear, highly fluctuating trend.

This study is of importance in the context of understanding the relationship between depression and age-related loss of cognitive function as it provides a dynamic view on how the declining cognition over time is mirrored in changes of depressive symptoms. Despite the well-established cross-sectional association between depressive symptoms and lower cognitive functions, the relation between depression and rate of cognitive decline is less clear, as some studies did not find an association^23^ or found an association only in some population sub-groups^24^. Even though our study detected a relatively small annual change in cognitive functioning in all depressive symptom groups, this decline has major importance on the population level. Previous studies demonstrated that even minor changes in cognition are strong predictors of lower quality of life^5^, decreased functioning^25^, development of dementia^3,18^ as well as mortality^2,26,27^.

Our study uniquely revealed that the character of the trend of declining cognitive functions is driven by the trajectory of depressive symptoms. Specifically, only individuals with constantly high as well as those with increasing depressive symptoms showed a sign of a linear trend. Few previous studies investigated the character of the trend of the declining cognitive functions. In the Yale Precipitating Events Project, Han and colleagues identified five trajectories of cognitive decline: none, minimal, moderate, progressive and rapid decline^28^. The moderate and progressive decline groups displayed an accelerating, non-linear trend^28^, while the pattern in other groups resembled a linear trend. While some similarities can be found between our results and the study by Han et al., the highly fluctuating trend we found in the constantly low depressive symptoms group and the decelerating trend present in the decreasing depressive symptoms group is not matching any of the trajectories identified by Han and colleagues. Neither is there any similarity with any of the three patterns of cognitive decline detected in the Cambridge City over 75 Cohort Study by Terrera and colleagues^29^. Given the robustness of our findings, we propose that non-linear strongly fluctuating patterns of cognitive decline are common and future studies should use sensitive modelling methods to reveal them.

Our study complements previous literature concerning the rate of cognitive decline depending on trajectories of depressive symptoms^17–19^. Using a cohort of more than 4000 middle-aged and older participants of the Korean Longitudinal Study of Ageing, Choi and colleagues identified trajectories of depressive symptoms as low, increasing, moderate declining and high. Diverging from our findings, they found the greatest magnitude of cognitive decline in the high depressive symptoms trajectory and the lowest decline in the low depressive symptoms trajectory^17^. On less than 2000 older adults from the Monongahela-Youghiogheny Healthy Aging Team, Graziane et al. revealed five depressive symptom trajectories: rarely depressed, low-grade decreasing, low-grade increasing, moderate-grade and higher-grade symptoms^19^. Surprisingly, the authors found that persistently low cognitive functioning was associated the strongest with the low-grade increasing and moderate-grade group, and not with the higher-grade symptoms group^19^.

In a study on over 7000 participants of The English Longitudinal Study of Ageing, three distinct groups based on trajectories of depressive symptoms were observed—none, episodic and persistent—and individuals with the persistent depressive symptoms were found to have the steepest decline in all domains of cognitive functioning^18^. Some studies addressed the relationship between depressive symptoms trajectory with a risk of dementia^30,31^. In a cohort of more than 2000 participants of the Health, Aging, and Body Composition study^30^, Kaup and colleagues found that individuals exhibiting high and increasing depressive symptoms trajectories are at higher risk for dementia than those with consistently minimal symptoms or moderate and increasing symptoms^30^. The results also indicated that the high and increasing trajectory was associated with dementia incidence, while depressive symptoms at individual time points were not. On more than 3000 participants of the Rotterdam study^31^, Mirza and colleagues observed significantly higher risk of dementia only in the trajectory of increasing depressive symptoms^32^.

There is an ongoing debate whether depression and cognitive decline in later life are results of common causes, such as cardiovascular risk factors and dysregulation of the hypothalamic–pituitary–adrenal axis, or whether depression causes cognitive decline or if it is the other way around^33^. We propose that there may be different biological mechanisms in place for each subgroup characterized by a unique trajectory of depressive symptoms. In the present study, we observed that individuals at the highest risk of rapid cognitive decline are those, in whom depressive symptoms increase late in life. This is in line with the view that depression is an early manifestation of cognitive disorders, rather than its cause^34^. The subgroup with increasing depressive symptoms may thus indicate people with underlying neurodegenerative pathologies and ongoing neuroinflammatory reaction. In a secondary analysis, we attempted to test, whether changes in depressive symptoms precede changes in cognition or vice versa by performing a test for Granger causality. This test indicated that changes in cognitive functioning precede changes in depressive symptoms, which supports the view that the occurrence of depressive symptoms is a consequence of cognitive deterioration. However, these results should be interpreted with caution as the chosen causal modelling approach may have not been complex enough to take into account all factors that could influence the relationship of depressive symptoms and cognitive functioning. A robust modelling approach, such as an autoregressive latent trajectory model, may be needed to fully address the directionality of depressive symptoms and cognition relationship^35^.

Even though the most likely causal explanation is that the emergence of depressive symptoms follows incipient cognitive decline, the strength and uniqueness of our analytical approach is that it gives insights into other mechanisms that may exist for smaller groups of the population. A different mechanism may, therefore, exist for the subgroup with the second highest rate of cognitive decline, which are individuals with constantly high depressive symptoms. Our finding supports the notion that depression in this subgroup may be causally linked to cognitive decline and could through neurotoxic effects cause harm to the brain that manifests with cognitive deficits. Especially long-term exposure to depression is thus particularly toxic to the brain and may lead to reduced neurogenesis^36^. Even though it may seem surprising that the level of cognitive decline in the constantly high depressive symptoms group does not reach the magnitude that is present in the sub-population with increasing depressive symptoms, it is analogous with findings from a study by Sachs-Ericsson and colleagues. They revealed steeper cognitive decline in individuals with late-onset depression (LOD) than in those with early-onset depression (EOD), even though the EOD group displayed more depressive symptoms at baseline that the LOD group^37^.

Two population groups in our study have relatively spared cognitive functioning over time. First, it is individuals with constantly low depressive symptoms. If we assume that cognitive decline precedes the occurrence of depressive symptoms, this result indicates that cognitive health highly contributes to mental health. Alternatively, this finding can also support the view that this subgroup of individuals was not exposed to neurotoxic effects of depression. Second, cognitive decline was low in individuals, in whom depressive symptoms were present at baseline, but decreased with time. We argue that these findings likely indicate that short term exposure to depression is not detrimental to long-term cognitive functioning. Once the depressive symptoms decreased, cognitive functioning improved, reaching levels closely resembling those before the occurrence of depressive symptoms. Even though mixed evidence exists with respect to whether cognitive skills improve after successful treatment of depression, with some authors suggesting that cognitive impairment is present even when depression is in remission^38–42^, our findings indicate that the treatment of depression could potentially reduce the rate of cognitive decline, underlying the importance of accurate diagnosis and therapy of depression in older adults.

Several limitations of this study need to be mentioned. First, as the distance between individual waves of the study was in years, we cannot rule out that we did not capture precisely enough the potential within-individual fluctuation of depressive symptoms over time. In addition, although the EURO-D measure was developed to be used specifically in the population of older adults, we cannot rule out the possibility that changes in cognitive functions could have influenced the reporting of depressive symptoms^43^. Second, participants of SHARE are in general healthier and more educated than the general population, which may underestimate the burden of depression as well as the magnitude of cognitive decline in our study. Third, the included cognitive measures (verbal fluency, immediate recall, and delayed recall) do not comprehensively reflect overall cognitive abilities. The present study thus only reflects verbal and memory functioning. Forth, although we assessed the directionality of depressive symptoms and cognition relationship in a secondary analysis, more robust causal modelling approaches may be required to fully uncover this relationship. On the other hand, this study has major strengths. To the best of our knowledge, our study used the largest sample to date to examine the association of depressive symptoms and cognitive decline. Another unique strength of this study is wide geographical representation of participants that reside in 18 countries. Furthermore, we used a robust data-driven approach to establish the trajectories of depressive symptoms, following established guidelines. This is preferable to approaches, in which the groups are created based on arbitrary criteria.

The findings that the individuals with decreasing depressive symptoms demonstrated the lowest rate of cognitive decline, is of significant public health interest as it potentially shows that reducing levels of depression could also lead to a reduction in the rate of cognitive decline. That, in turn, could lead to postponing the onset of cognitive impairment and dementia, thus considerably lowering the associated burden of disease. The possibilities of pharmacotherapy, psychotherapy or multimodal therapy focused on lowering depressive symptoms and/or depression in older adults should be further investigated. Moreover, as the greatest change in cognitive functioning was detected in individuals with increasing depressive symptoms, more research should be conducted on assessing the precise characteristics of these individuals. That may, then, enable more tailored interventions, with a large potential to delay the development of cognitive impairment and/or dementia.

## Methods

### Source of data

Data for the analysis were acquired within a prospective cohort study Survey on Health, Ageing and Retirement in Europe (SHARE), a pan-European study of health, social network and economic conditions of community-dwelling individuals that was previously described in detail^44^. Briefly, participants were sampled based on probability selection methods. Individuals eligible for the study were people aged 50 years and older and their spouses, irrespective of age. Data was collected using computer-assisted personal interviewing (CAPI) in participants’ homes. The study was initiated in 2004, followed by five subsequent waves in approximately two-year intervals, with wave 7 completed in 2017.

This study was carried out in accordance with the Declaration of Helsinki. SHARE has been repeatedly reviewed and approved by the Ethics Committee of the University of Mannheim. All participants provided a written informed consent. Data were pseudo-anonymized and participants were informed about the storage and use of the data and their right to withdraw consent. The present analysis was approved by the Ethics Committee of the National Institute of Mental Health, Czech Republic.

### Depressive symptoms

Depressive symptoms were assessed in waves 1, 2, 4, 5, 6 and 7, using the EURO-D scale^45^, which is a self-assessment tool to measure symptoms of depression in older adults across Europe and has been used in many previous studies^46–48^. The EURO-D scale consists of 12 items (depressed mood, pessimism, wishing death, guilt, sleep, interest, irritability, appetite, fatigue, concentration, enjoyment, and tearfulness), which are scored 0 (symptom not present) or 1 (symptom present), with the reference period being the last month. This generates a simple scale ranging between 0 and 12, with higher values indicating higher risk of depression.

### Cognition

Cognition was assessed in wave 1, 2, 4, 5, 6 and 7 using three measures: verbal fluency, immediate recall and delayed recall. Verbal fluency score was derived from an animal fluency test and indicates the sum of acceptable animals that the participants could name within one minute. Immediate and delayed recall were extracted from an adapted 10-word delay recall test^49^. Immediate recall score (range 0–10) was the number of recalled words after the interviewer read a list of 10 words from their computer screen. At the end of the cognitive testing session, the participants were asked again to recall any of the words from the list, which captured delayed recall score (range 0–10). To avoid practice effects, different versions of the cognitive tests were used in each wave. In this manuscript, we are referring to these three measures as “cognitive functions” and “cognitive decline”.

### Covariates

Information on sociodemographic and health-related characteristics, as potentially confounding and mediating factors in the association of depressive symptoms and cognitive decline, were initially identified based on literature^50–52^. The selected sociodemographic factors were age (years), sex (men vs. women), education (categories based on the International Standard Classification of Education 1997^53^), household net worth (standardized difference between household gross financial assets and financial liabilities), current job situation (retired vs. employed vs. unemployed vs. permanently sick vs. homemaker), family status (married and living with spouse vs. registered partnership vs. married and not living with spouse vs. never married vs. divorced vs. widowed), number of children and grandchildren (irrespective of them being biological, adopted or step-children).

Health-related characteristics were treatment for depression (defined by self-reported information on the use of drugs against depression or anxiety), number of limitations in instrumental activities of daily living (IADL), number of chronic diseases, body mass index, physical inactivity (never vigorous nor moderate physical activity vs. physical activity), smoking (ever smoked daily vs. never smoked daily), alcohol use (more than 2 glasses of alcohol almost every day vs. less), and maximal grip strength. In the case of time-variant variables (BMI, physical inactivity, maximal grip strength, household net worth, marital status, number of children, number of grandchildren, current job status, IADL, number of chronic diseases, treatment for depression), we have retrieved them per every wave of study separately.

### Trajectories of depressive symptoms

We generated trajectories of depressive symptoms using latent growth mixture modelling, a constrained exploratory approach that differentiates unobserved groups of individuals based on their probability of following a similar trajectory on an outcome over time. Model selection procedures were guided according to recommended procedures^54^. For a fully detailed description of the performed procedures see Supplement. A latent basis growth model (LBGM) with freely estimated slopes at each time point was entered into the latent growth mixture model.

**Figure 4:**
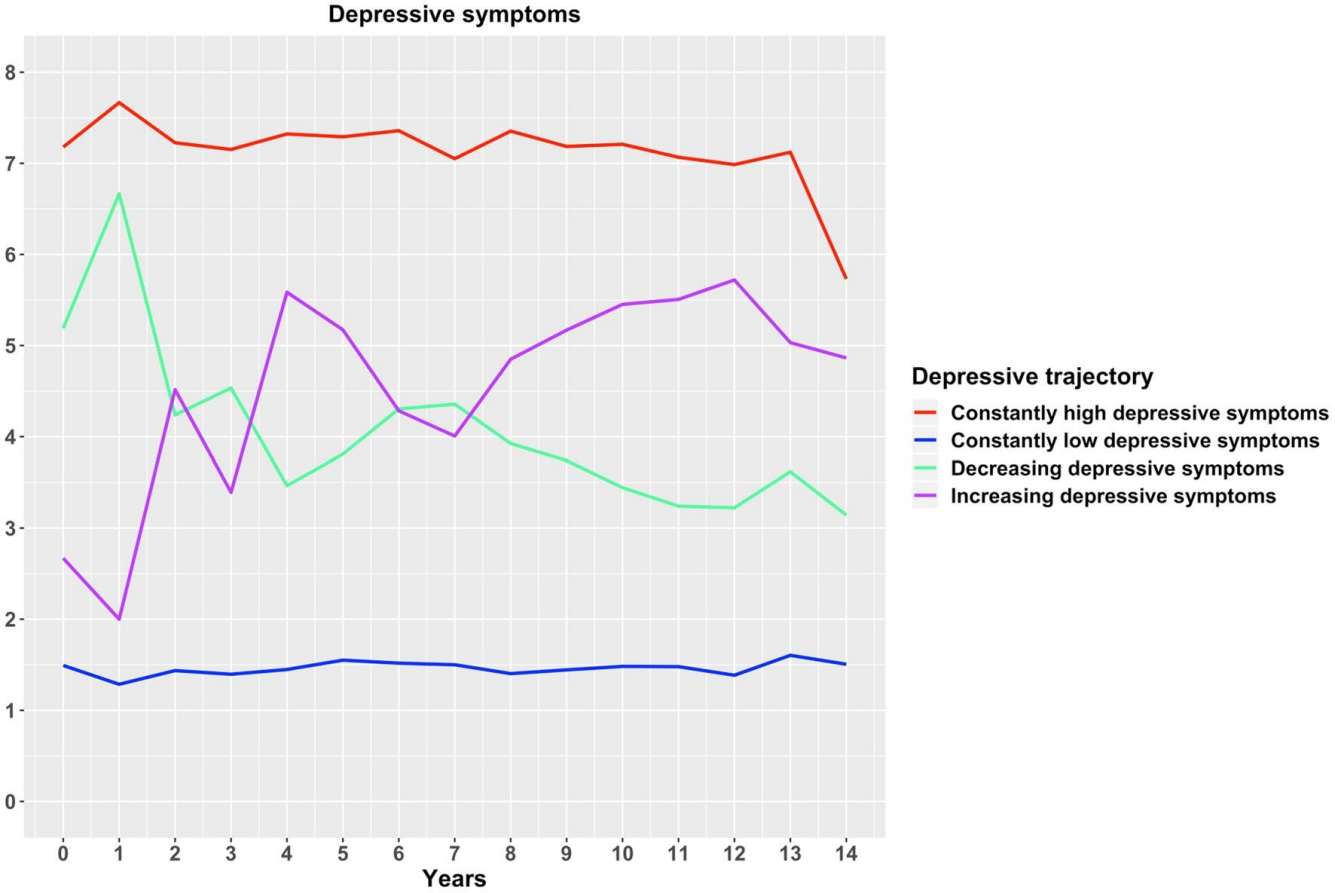
Trajectories of depressive symptoms.

The model with the best supporting criteria and the best interpretation was the 4-class model (cf. mean depressive symptoms across the latent trajectories in each of the 1-, 2-, 3-, 4-, 5-, 6-, 7-, and 8-class solution along each time of measurement in the Figure S2). The selected 4-class model for the subsequent analyses was the following: Class 1) Constantly high depressive symptoms (4.3%), Class 2) constantly low depressive symptoms (71.9%), Class 3) decreasing depressive symptoms (13.9%) and Class 4) increasing depressive symptoms (9.9%), see Fig. 4. These categories are data-driven and do not necessarily correspond to the presence or lack of clinical diagnoses of a major depressive episode. In previous studies, the cut-off of four symptoms on the EURO-D scale was used to indicate the presence of major depression^46,50^. If we applied this criterion to our trajectories, Class 1 would correspond to the constantly present major depressive episode, Class 2 to the constantly absent major depressive episode, Class 3 to a transition from a major depressive episode towards subthreshold depression and Class 4 to a transition from subthreshold depression towards a major depressive episode. The baseline covariates were entered into the 4-class model predicting the latent class variable in a multinomial regression using the 3-step method. Entering the covariates into the model still allowed to replicate the results of the 4-class model.

### Analytical sample

We restricted the analysis to participants in SHARE older than 50 years at baseline that had at least two measures of depressive symptoms and two measures of cognition (baseline is considered the first time at least one cognitive measure was recorded). From 139,556 individuals that completed at least 1 interview in SHARE, we excluded 49,301 people who participated only in 1 interview. We then excluded 17,266 individuals who did not have data on depressive symptoms in at least 2 waves and further 1056 individuals who did not have data on all three cognitive measures in at least 2 waves. If a participant had data on all cognitive tests in at least 2 waves, then they were allowed to have incomplete data on cognitive tests on subsequent waves.

We then excluded 2497 individuals younger than 50 years. In addition, we also excluded 370 people who did not have the measure of depressive symptoms at baseline in the same wave as cognition. The final analytical sample consisted of 69,066 people (on average 64 years at baseline, 55% women), from whom 56% had at least 3 measures of EURO-D and verbal fluency and 77% had at least 3 measures of immediate recall and delayed recall. The participants were followed up for the median of 6 years (range 1–12 years) since baseline. The flowchart is presented on Supplemental Figure S1.

### Statistical analysis

Once participants were divided into trajectories of depressive symptoms, we used descriptive statistics (Chi-square test, analysis of variance and Kruskall Wallis test) to evaluate whether they differ in baseline sociodemographic and health-related characteristics. In addition, we applied multinomial logistic regression to investigate the association of all characteristics with groups of depressive symptoms (using individuals with constantly low depressive symptoms as reference).

We employed smoothing splines mixed effects models (with p-spline basis) to estimate the course of cognitive decline for each trajectory of depressive symptoms. These models allow for modelling a wide range of non-linear behaviors and we used them to visualize the patterns of cognitive decline per trajectories of depressive symptoms. Furthermore, we used linear mixed effects models, with individuals set as random intercepts and time (in years) between waves of study as random slope, to estimate the rate of cognitive decline, stratifying the data based on each trajectory of depression. We stratified the data instead of including an interaction of depressive trajectories and time to obtain a separate coefficient for every trajectory. Moreover, based on the fitted models adjusted for baseline, sex, education and country of origin, we visualized the cognitive decline per trajectories of depressive symptoms. We performed this operation using raw scores, and to be able to compare the rates of cognitive decline between domains of cognitive functioning, we also used z-score transformed scores on individual measures of cognitive functioning. The z-scores standardization was performed per every measure for each wave of study separately. As our primary interest was to assess the rate of cognitive decline per depressive symptom trajectories, throughout the manuscript, we interpret only the results from models adjusted for baseline age, sex, education and country of origin. Additional adjustment for covariates would introduce too much complexity to the models and their interpretation.

We conducted three sets of secondary analyses. First, we divided the participants according to baseline age into two groups: younger than 65 years and 65 and older. Then, we fitted linear mixed effects models stratified by age group and depressive trajectory for all three cognitive domains, adjusting for baseline age and sex. Second, we classified the participants based on their region of residence. We created five regions: 1. CEE (Czechia, Poland, Slovenia, Estonia), 2. Scandinavia (Denmark, Sweden), 3. Southern Europe (SE, Spain, Greece, Italy, Portugal), 4. Western Europe (WE, Austria, Germany, Belgium, Switzerland, France, Netherlands, Luxemburg) and 5. Israel. We, then, employed linear mixed effects models to assess the rate of cognitive decline stratified by regions and depressive trajectory, adjusting for baseline age and sex. Third, we performed a test for Granger causality in order to assess whether changes in depressive symptoms precede changes in cognition or vice versa (see Supplementary Methods). Analyses were performed using R statistical programming language (version 3.6.0) and Mplus (version 8.4). Associations with *p* < 0.05 were considered as statistically significant.

## Supplementary information

Supplementary Information.

## Data Availability

Access to the SHARE data is provided free of charge on the basis of a release policy that gives quick and convenient access to all scientific users worldwide after individual registration. All details about the application and registration process can be found on this website: share-project.org. The study protocol and syntax of the statistical analysis will be shared upon request from the corresponding author of this study.
