# Supplementary material for: Trajectories of depressive symptoms and associated patterns of cognitive decline
Source: Sci Rep. 2020 Nov 30;10:20888. doi: 10.1038/s41598-020-77866-6 (PMC7705007; doi:10.1038/s41598-020-77866-6)
Supplement: Supplementary file 1 — Supplementary Information. [file 41598_2020_77866_MOESM1_ESM.docx]

**Trajectories of depressive symptoms and associated patterns of cognitive decline**

Tomáš Formánek^1^, MSc; Zsófia Csajbók^1,2^, MA; Katrin Wolfová^3^, MD; Matěj Kučera^1,3^; Sarah Tom, PhD^4^; Prof. Dag Aarsland^5,6^, MD, PhD; Pavla Cermakova^1,3,7^, MD, PhD*

1 National Institute of Mental Health, Klecany, Czech Republic

2 Faculty of Science, Charles University Prague, Czech Republic

3 Third Faculty of Medicine, Charles University, Prague, Czech Republic

4 Departments of Neurology and Epidemiology, Columbia University, New York, United States

5 Department of Old Age Psychiatry Institute of Psychiatry, Psychology & Neuroscience, King's College London, London, United Kingdom

6 Centre of Age-Related Medicine, Stavanger, University Hospital Stavanger, Norway

7 Second Faculty of Medicine, Charles University, Prague, Czech Republic

Correspondence to:

Pavla Cermakova, MD, PhD

National Institute of Mental Health

Topolová 748

250 67 Klecany

Czech Republic

Pavla.Cermakova@nudz.cz

**SUPPLEMENT**

**Supplementary methods**

*Latent growth mixture modelling*

We generated trajectories using latent growth mixture modelling, a constrained exploratory approach that differentiates unobserved groups of individuals based on their probability of following a similar trajectory on an outcome over time. Model selection procedures were guided according to recommended procedures . Missing values have been estimated using the default Mplus procedure: Full information maximum likelihood method (FIML). First, we compared three latent growth models’ (LGM) fit against the data before the classification: an LGM with latent intercept and slope factors (fixed to test linear growth with fixed slopes using the first wave of the study as time 0, the second as time 2, the fourth as time 6, the fifth as time 8, the sixth as time 10, and the seventh as time 12, as the participants were tested on depressive symptoms every two years except in the third wave); latent growth curve model (LGCM) with latent intercept, slope and quadratic factor (with fixed slopes similar to the linear growth model); and a latent basis growth model (LBGM) with only intercept and slope factors but with freed slopes estimating the rate and shape of change completely freely (with the first time of measurement fixed at zero, and the last fixed at one). The best model fit was yielded by the LGCM but the estimated mean of the quadratic factor was only -0.001 (p < .001; cf. Supplementary table 4). The LBGM model with freely estimated slopes in each time point was the second best fitting model with strong coefficients, thus we entered the LBGM model into the latent growth mixture model since it had comparable – if not better – abilities in detecting non-linear (and any shape of) growth within the identified latent trajectories.

Entering the freely estimated latent base growth model into the latent growth mixture modelling (LGMM), we extracted 2-, 3-, 4-, 5-, 6-, 7-, and 8-class models in each model predicting the respective number of latent trajectories. The variance of the growth parameters was estimated in each model and constrained to be equal across the latent trajectories as default in Mplus. Each classification model was rerun using different random starts at least 3 times and fourth time using the LRT-starts option to yield reliable parametric bootstrapped likelihood ratio tests for each model. The best classification model was chosen based on considering a number of factors. Each model has been successfully replicated except for the 8-class model which could not yield non-negative latent variable covariance matrix even with the random starts increased to 10 000. The best log-likelihood values were replicated within each model runs and between each run with different sets of random starts. The Akaike Information Criterion (AIC), consistent Akaike Information Criterion (CAIC), Bayesian Information Criterion (BIC), and sample-size adjusted Bayesian Information Criterion (SSA BIC) were all replicated in each model which was started with different sets of random starts; and improved with each additional latent trajectory.

The entropy was acceptable in each model, though decreased with each additionally extracted class. The Vuong-Lo-Mendell-Rubin Likelihood Ratio Test (VLMR LRT) was significant in each model (p < 0.001) indicating a better model in the k-class models versus in the k-1 class models (with a significant test indicating better k-class model in comparison to the k-1 class solution). Similarly to the VLMR-LRT test, the Luo-Mendell-Rubin adjusted likelihood ratio test (LMR ALRT) also recommended the k-class models in comparison to the k-1 class models in each solution (p < 0.001; even in the 8-class solution). Finally, the parametric bootstrapped likelihood test also favored the k-class solutions in contrast to the k-1 class models with significant approximate p values (p < 0.001, Supplementary table 5). Therefore, as all the solutions have been supported by the criteria except for the 8-class solution, we chose the final model for subsequent analyses based on their interpretability. The model with the best interpretation was the 4-class model (Supplementary table 2; cf. estimated mean depressive symptoms across the latent trajectories in each of the 1-, 2-, 3-, 4-, 5-, 6-, 7-, and 8-class solution along each time of measurement in the Supplementary figures 2a-h). The freely estimated slopes in the final, 4‑class model can be found in Supplementary table 3.

The following 4-class model was selected for the analysis (with proportion of participants): 1) Constantly high depressive symptoms (4.3%), 2) constantly low depressive symptoms (71.9%), 3) decreasing depressive symptoms (13.9%) and 4) increasing depressive symptoms (9.9%). Although some recommend to keep solutions of the classification only if all the identified classes contain at least 5% cases of the total sample, in our results the class having constantly high depressive symptoms still had sufficient number of cases for further analyses (N=2 998) even though proportionally the class may seem small. Also, the sub-population having constantly high depressive symptoms, from a theoretical point of view, is likely to be only a fraction of the population. Due to the low covariance coverage in the total sample which may have biased the analyses, we performed the 4-class LGMM on a subset of the sample where each participant had data available on their depressive symptoms in each time of measurement (N=4 899). The 4-class solution on the fully covered sample was interpretatively comparable to the one on the large sample, and practically the same, supporting the results using the FIML method on the large sample (Supplementary figure 3). The baseline covariates were entered into the 4-class model predicting the latent class variable in a multinomial regression using the 3-step method (Supplementary table 1). Entering the covariates into the model still allowed to replicate the results of the 4-class model.

*Granger causality*

To assess whether changes in cognitive functioning precede changes in depressive symptoms or vice versa, we performed a test for Granger causality. First, we averaged the scores on cognitive tests and EURO-D across all of the time points, expressed in months and excluding baseline values (time zero). Thus, we obtained averages corresponding to 127 time points. Second, we created a time series for every cognitive test and EURO-D combination separately. Third, we assessed stationarity using the augmented Dickey–Fuller test and determined the number of lags to achieve stationarity. In every case, the number of differences to achieve stationary series was equal to one. Forth, we determined the optimal number of lags for the Granger causality test using the Akaike Information Criterion (AIC), Schwarz Criterion (SC), Hannan-Quinn (HQ) Criterion and Final Prediction Error (FPE). The number of lags was unanimously determined to be equal to one on both, immediate and delayed recall test. On the verbal fluency test, it was determined as either one or five, so we decided to use one to be in-line with other tests.

**Supplementary tables**

**Supplementary table 1** Associations of participants´ characteristics with trajectories of depressive symptoms

| Depressive symptoms | | | | |  |
| --- | --- | --- | --- | --- | --- |
|  | Constantly low | Constantly high | Increasing | Decreasing | |
| Age | Reference | 0.97 (0.97; 0.98)** | 1 (1; 1.01) | 0.98 (0.98; 0.99)** | |
| Woman |  | 1.1 (0.96; 1.25) | 1.31 (1.2; 1.42)** | 1.28 (1.19; 1.38)** | |
| More than high school education |  | 0.44 (0.39; 0.51)** | 0.72 (0.67; 0.77)** | 0.72 (0.67; 0.76)** | |
| Highest decile of household net worth |  | 0.58 (0.48; 0.71)** | 0.83 (0.76; 0.92)** | 0.71 (0.65; 0.78)** | |
| Living with a partner |  | 0.79 (0.72; 0.87)** | 1 (0.94; 1.06) | 0.8 (0.76; 0.84)** | |
| 2 and more children |  | 0.9 (0.81; 1)* | 0.97 (0.91; 1.04) | 0.94 (0.88; 0.99)* | |
| 2 and more grandchildren |  | 1.1 (0.99; 1.21) | 1.04 (0.98; 1.11) | 1.05 (0.99; 1.11) | |
| Currently working |  | 0.79 (0.71; 0.87)** | 0.86 (0.81; 0.92)** | 0.86 (0.82; 0.91)** | |
| Treatment of depression |  | 7.03 (6.29; 7.86)** | 2.58 (2.32; 2.86)** | 3.44 (3.16; 3.75)** | |
| Body mass index |  | 1.01 (1; 1.02)* | 1.02 (1.02; 1.03)** | 1.01 (1; 1.01)** | |
| 2 and more chronic diseases |  | 3.05 (2.76; 3.36)** | 1.55 (1.47; 1.64)** | 2.04 (1.94; 2.14)** | |
| 2 and more limitations in IADL |  | 4.21 (3.71; 4.77)** | 1.75 (1.55; 1.98)** | 2.66 (2.4; 2.94)** | |
| Maximal grip strength |  | 0.93 (0.93; 0.94)** | 0.97 (0.97; 0.98)** | 0.96 (0.96; 0.97)** | |
| Physical inactivity |  | 2.68 (2.4; 2.99)** | 1.58 (1.44; 1.73)** | 1.8 (1.67; 1.95)** | |
| Smoking |  | 1.05 (0.96; 1.15) | 1 (0.95; 1.06) | 1.12 (1.06; 1.17)** | |
| Alcohol |  | 1.04 (0.91; 1.19) | 1.12 (1.04; 1.21)* | 1.07 (1; 1.15)* | |

Results are odds ratio with 95% confidence intervals derived from multinomial logistic regression for the associations of participants´ characteristics with groups of individuals with significant depressive symptoms, compared to individuals who never had significant depressive symptoms. All participants´ characteristics were entered into the model simultaneously.

*p<0.05; **p<0.001; OR, odds ratio; CI, confidence interval

**Supplementary table 2** Class proportions and mean intercept and slope results in the 4-class latent base growth model

|  | | | | |
| --- | --- | --- | --- | --- |
|  | **N of class members** | **% of total N** | **i (S.E.)** | **s (S.E.)** |
| **Class 1** | 2998 | 4.341 | 6.868 (0.088)*** | 0.152 (0.120) |
| **Class 2** | 6829 | 9.888 | 2.300 (0.091)*** | 2.330 (0.141)*** |
| **Class 3** | 9579 | 13.869 | 5.011 (0.130)*** | -1.364 (0.128)*** |
| **Class 4** | 49660 | 71.902 | 1.467 (0.022)*** | -0.024 (0.019) |
| S.E. = standard error of the mean.  *** p < 0.001. | | | | |

**Supplementary table 3** Estimated slopes in the 4-class latent base growth model

|  | |
| --- | --- |
|  | **Estimates of slopes (S.E.)** |
| **EURO-D Wave 1** | 0.000 (0.000) |
| **EURO-D Wave 2** | 0.016 (0.061) |
| **EURO-D Wave 4** | 0.342 (0.037)*** |
| **EURO-D Wave 5** | 0.635 (0.034)*** |
| **EURO-D Wave 6** | 1.522 (0.082)*** |
| **EURO-D Wave 7** | 1.000 (0.000) |
| S.E. = standard error of the mean.  *** p < 0.001. | |

|  | | | | | | | | | | | | |
| --- | --- | --- | --- | --- | --- | --- | --- | --- | --- | --- | --- | --- |
| **Model** | **Estimated** | **Slopes** | **X^2^(df)** | **RMSEA** | **p-close** | **CFI** | **TLI** | **SRMR** | **AIC** | **CAIC** | **BIC** | **SSA BIC** |
| 1-class latent growth | i s | fixed (linear) | 515.328(16) | 0.021 | [.020; .023] | 0.990 | 0.991 | 0.025 | 874789.823 | 874901.394 | 874890.394 | 874855.436 |
| 1-class latent growth curve | i s q | fixed quadratic | 276.798(12) | 0.018 | [.016; .020] | 0.995 | 0.994 | 0.015 | 874559.293 | 874711.435 | 874696.435 | 874648.764 |
| 1-class latent base growth | i s | freed | 423.196(12) | 0.022 | [.020; .024] | 0.992 | 0.990 | 0.021 | 874705.691 | 874857.833 | 874842.833 | 874795.163 |
| *Note.* RMSEA=root mean square of approximation; CFI=comparative fit index; TLI=Tucker-Lewis index; AIC=Akaike information criterion; CAIC=consistent Akaike information criterion; BIC=Bayesian information criterion; SSA BIC=sample-size adjusted Bayesian information criterion. | | | | | | | | | | | | |

**Supplementary table 4** Model fit of the 1-class latent growth model with fixed linear slopes; the 1-class latent growth curve model; and the 1-class latent base growth model with freed slopes on the total sample

**Supplementary table 5** Model parameters and results of the 2-, 3-, 4-, 5-, 6-, 7-, and 8-class latent base growth models

|  | | | | | | | | | |
| --- | --- | --- | --- | --- | --- | --- | --- | --- | --- |
| **N of classes** | **Best Log-Likelihood** | **AIC** | **CAIC** | **BIC** | **SSA BIC** | **Entropy** | **VLM RLR** | **LMR ALRT** | **BLRT** |
| 2 | -432125.707 | 864287.413 | 864469.984 | 864451.984 | 864394.779 | 0.793 | p < 0.001 | p < 0.001 | p < 0.001 |
| 3 | -429550.487 | 859142.974 | 859289.235 | 859334.974 | 859268.235 | 0.774 | p < 0.001 | p < 0.001 | p < 0.001 |
| 4 | -427905.759 | 855859.518 | 856102.945 | 856078.945 | 856002.673 | 0.744 | p < 0.001 | p < 0.001 | p < 0.001 |
| 5 | -426759.974 | 853573.948 | 853847.804 | 853820.804 | 853734.998 | 0.733 | p < 0.001 | p < 0.001 | p < 0.001 |
| 6 | -425960.210 | 851980.421 | 852284.705 | 852254.705 | 852159.365 | 0.73 | p < 0.001 | p < 0.001 | p < 0.001 |
| 7 | -425365.392 | 850796.783 | 851131.496 | 851098.496 | 850993.622 | 0.717 | p < 0.001 | p < 0.001 | p < 0.001 |
| 8^a^ | -424768.777 | 849609.555 | 849974.696 | 849938.696 | 849824.287 | 0.721 | p < 0.001 | p < 0.001 | p < 0.001 |
| *Note.* AIC=Akaike information criterion; CAIC=consistent Akaike information criterion; BIC=Bayesian information criterion; SSA BIC=sample-size adjusted Bayesian information criterion; VLM RLT=Vuong-Lo-Mendell-Rubin likelihood ratio test; LMR ALRT=Luo-Mendell-Rubin adjusted likelihood ratio test; BLRT=bootstrap likelihood ratio test.  ^a^ Negative variance of the latent slopes. | | | | | | | | | |

**Supplementary table 6** Cognitive decline using linear mixed effects models across trajectories of depresssive symptoms stratified by baseline age

|  | Depressive symptoms | | | |
| --- | --- | --- | --- | --- |
|  | Constantly high | Constantly low | Increasing | Decreasing |
| Immediate recall |  |  |  |  |
| Baseline age < 65 | -0.00 (-0.02; 0.01) | -0.00 (-0.00; 0.00) | -0.03 (-0.03; -0.02)*** | 0.01 (0.00; 0.02)*** |
| Baseline age >= 65 | -0.08 (-0.09; -0.06)*** | -0.05 (-0.05; -0.05)*** | -0.09 (-0.10; -0.08)*** | -0.05 (-0.06; -0.04)*** |
| Delayed recall |  |  |  |  |
| Baseline age < 65 | -0.01 (-0.02; 0.00) | 0.01 (0.01; 0.01)*** | -0.03 (-0.04; -0.02)*** | 0.02 (0.01; 0.03)*** |
| Baseline age >= 65 | -0.07 (-0.08; -0.06)*** | -0.05 (-0.05; -0.04)*** | -0.09 (-0.10; -0.08)*** | -0.05 (-0.06; -0.04)*** |
| Verbal fluency |  |  |  |  |
| Baseline age < 65 | -0.10 (-0.15; -0.05)*** | -0.02 (-0.03; -0.01)*** | -0.15 (-0.19; -0.12)*** | -0.02 (-0.04; 0.01) |
| Baseline age >= 65 | -0.32 (-0.37; -0.28)*** | -0.20 (-0.22; -0.19)*** | -0.39 (-0.42; -0.36)*** | -0.23 (-0.26; -0.20)*** |

Results are β (95% CI) derived from linear mixed-effects models

*p<0.05; **p<0.01; ***p<0.001

All models were adjusted for baseline age, sex, education and country of origin.

**Supplementary table 7** Cognitive decline using linear mixed effects models across trajectories of depresssive symptoms stratified by regions

|  | Depressive symptoms | | | |
| --- | --- | --- | --- | --- |
|  | Constantly high | Constantly low | Increasing | Decreasing |
| Immediate recall |  |  |  |  |
| CEE | -0.05 (-0.07; -0.03)*** | -0.02 (-0.03; -0.01)*** | -0.07 (-0.08; -0.05)*** | -0.02 (-0.03; -0.01)*** |
| Israel | -0.05 (-0.09; -0.02)* | 0.00 (-0.01; 0.01) | -0.03 (-0.06; -0.00)* | 0.00 (-0.02; 0.03) |
| Scandinavia | -0.09 (-0.13; -0.05)*** | -0.04 (-0.04; -0.04)*** | -0.08 (-0.10; -0.07)*** | -0.04 (-0.05; -0.02)*** |
| SE | -0.03 (-0.05; -0.02)*** | -0.01 (-0.02; -0.01)*** | -0.06 (-0.07; -0.05)*** | -0.01 (-0.02; -0.00)* |
| WE | -0.03 (-0.05; -0.02)*** | -0.01 (-0.02; -0.01)*** | -0.05 (-0.06; -0.04)*** | -0.01 (-0.02; -0.00)*** |
| Delayed recall |  |  |  |  |
| CEE | -0.06 (-0.08; -0.04)*** | -0.01 (-0.02; -0.01)*** | -0.07 (-0.08; -0.05)*** | -0.01 (-0.03; -0.00)* |
| Israel | -0.06 (-0.09; -0.02)*** | 0.04 (0.03; 0.05)*** | -0.03 (-0.06; -0.00)* | 0.00 (-0.02; 0.03) |
| Scandinavia | -0.08 (-0.12; -0.04)*** | -0.03 (-0.04; -0.03)*** | -0.08 (-0.10; -0.06)*** | -0.03 (-0.05; -0.02)*** |
| SE | -0.05 (-0.06; -0.03)*** | -0.02 (-0.03; -0.02)*** | -0.08 (-0.09; -0.07)*** | -0.02 (-0.03; -0.01)*** |
| WE | -0.01 (-0.03; 0.00) | 0.00 (-0.00; 0.00) | -0.03 (-0.04; -0.02)*** | 0.01 (-0.00; 0.02) |
| Verbal fluency |  |  |  |  |
| CEE | -0.24 (-0.32; -0.16)*** | 0.00 (-0.03; 0.03) | -0.28 (-0.35; -0.22)*** | -0.04 (-0.09; 0.01) |
| Israel | -0.30 (-0.43; -0.17)*** | -0.01 (-0.06; 0.04) | -0.24 (-0.37; -0.11)*** | -0.14 (-0.24; -0.04)* |
| Scandinavia | -0.35 (-0.53; -0.17)*** | -0.12 (-0.14; -0.10)*** | -0.35 (-0.42; -0.28)*** | -0.19 (-0.25; -0.13)*** |
| SE | -0.19 (-0.24; -0.14)*** | -0.12 (-0.14; -0.11)*** | -0.28 (-0.31; -0.24)*** | -0.15 (-0.18; -0.11)*** |
| WE | -0.25 (-0.31; -0.19)*** | -0.11 (-0.12; -0.09)*** | -0.26 (-0.30; -0.22)*** | -0.11 (-0.15; -0.08)*** |

Results are β (95% CI) derived from linear mixed-effects models. All models were adjusted for baseline age, sex and education.

*p<0.05; **p<0.01; ***p<0.001

Central and Eestern Europe (CEE): Czechia, Poland, Slovenia, Estonia

Scandinavia: Denmark, Sweden

Southern Europe (SE): Spain, Greece, Italy, Portugal

Western Europe (WE): Austria, Germany, Belgium, Switzerland, France, Netherlands, Luxemburg

**
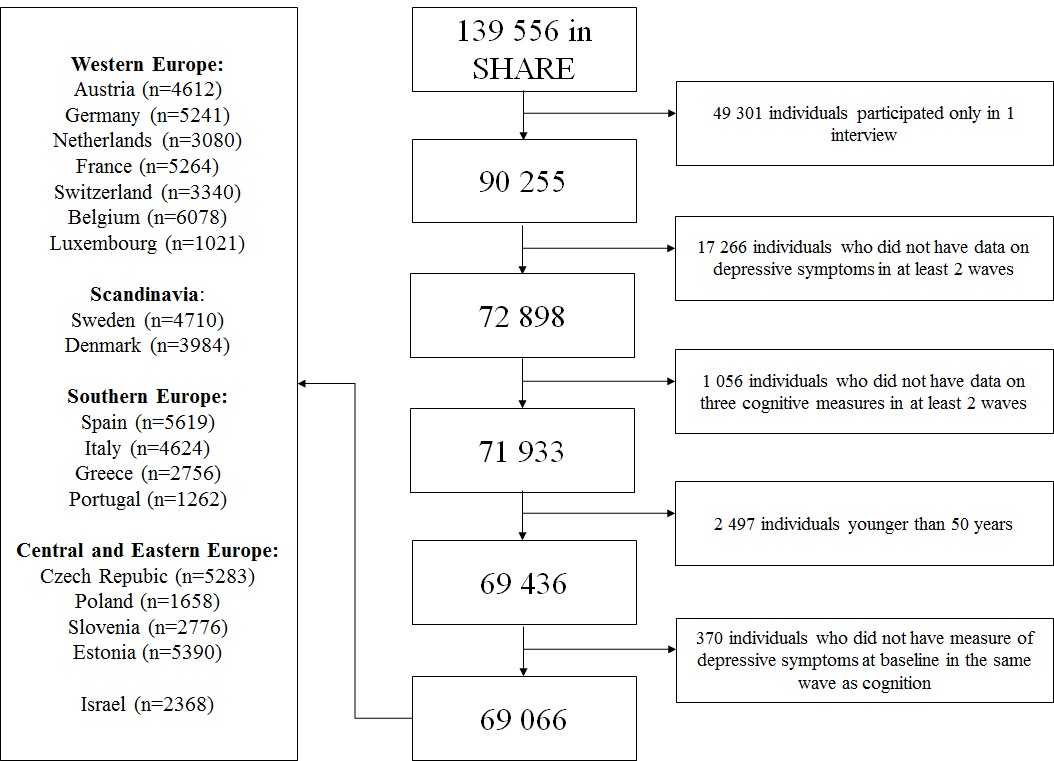
Supplementary figures**

**Figure S1** Selection of study participants

| 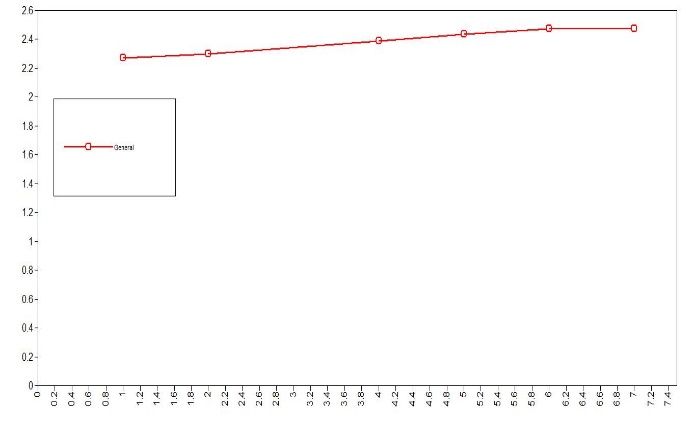  **A** | 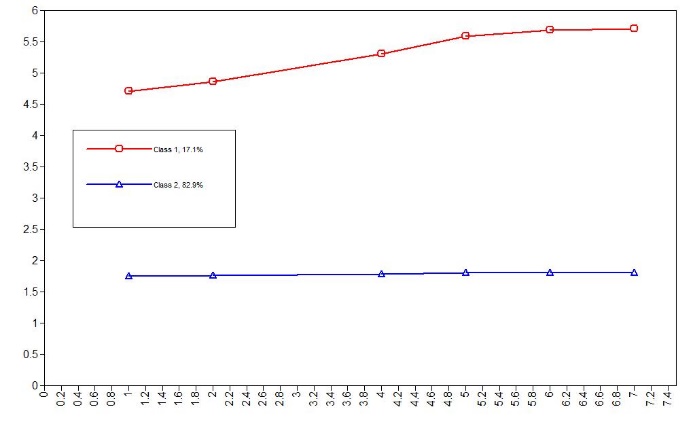  **B** |
| --- | --- |
| 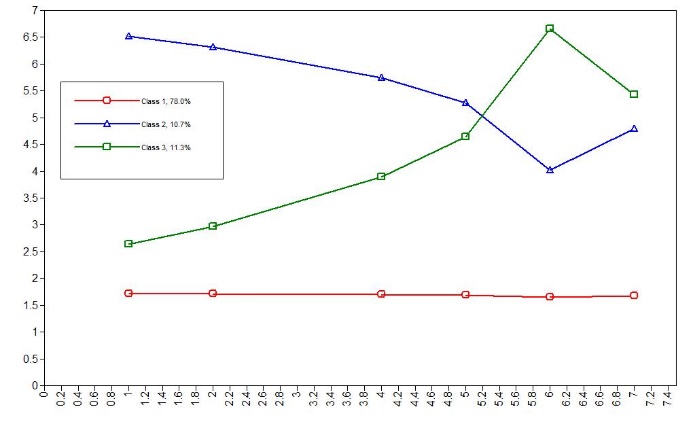  **C** | 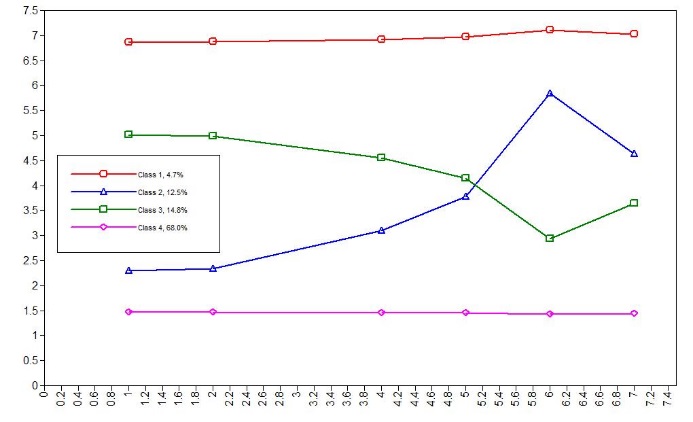  **D** |
| 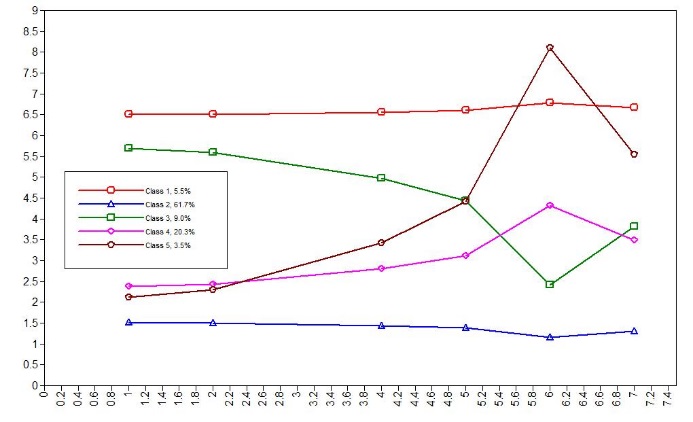  **E** | 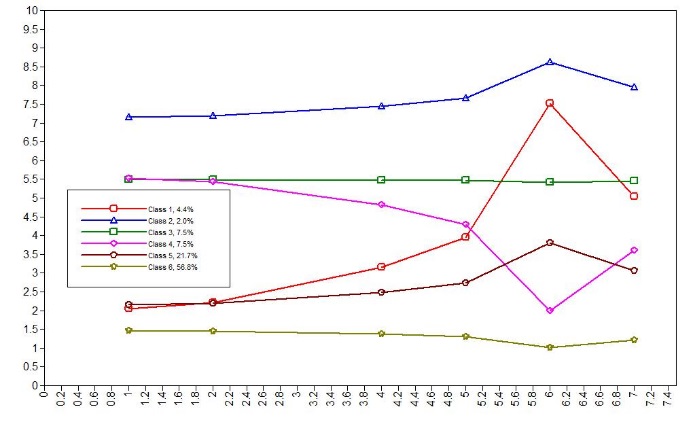  **F** |
| 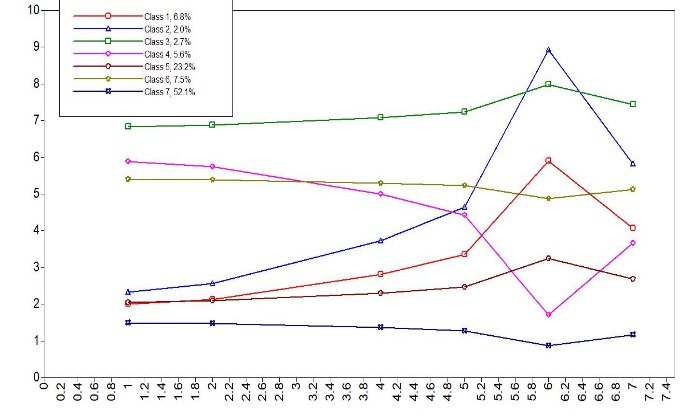  **G** | 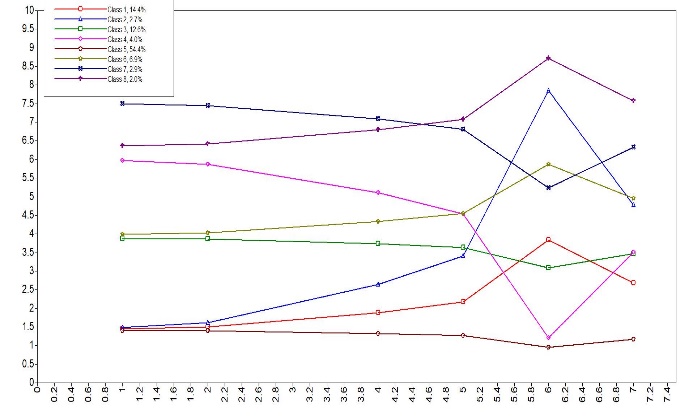  **H** |

**Figure S2** Mean depressive symptoms in different models

A 1‑class model; B 2‑class model; C 3‑class model; D 4‑class model; E 5‑class model; F 6‑class model; G 7‑class model; H 8‑class model


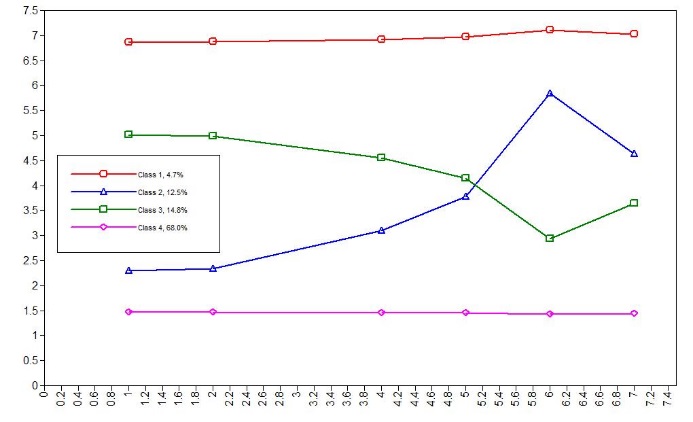

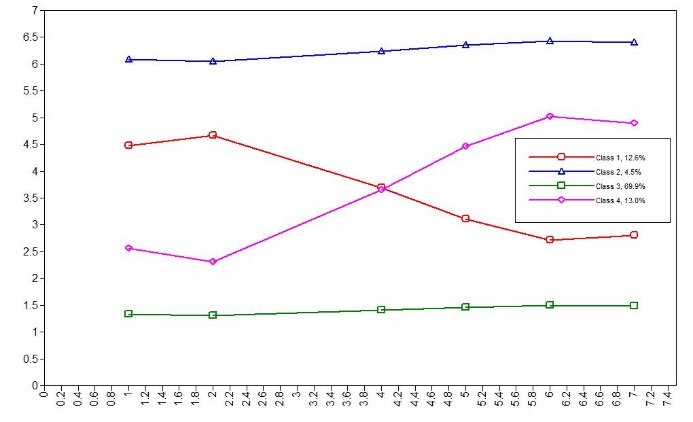


|  |  |
| --- | --- |
| References: | |

**Figure S3** Qualitative comparison between the results of the 4-class model estimated on the total sample (on the left; N=69 066) versus on the subsample having full data coverage in all 6 waves (on the right; N=4 899)

1 van de Schoot, R., Sijbrandij, M., Winter, S. D., Depaoli, S. & Vermunt, J. K. The GRoLTS-Checklist: Guidelines for Reporting on Latent Trajectory Studies. *Structural Equation Modeling: A Multidisciplinary Journal* **24**, 451-467, doi:10.1080/10705511.2016.1247646 (2017).
